# Acute myeloid leukemia cells require 6-phosphogluconate dehydrogenase for cell growth and NADPH-dependent metabolic reprogramming

**DOI:** 10.18632/oncotarget.18797

**Published:** 2017-06-28

**Authors:** Haymanti Bhanot, Ellen L. Weisberg, Mamatha M. Reddy, Atsushi Nonami, Donna Neuberg, Richard M. Stone, Klaus Podar, Ravi Salgia, James D. Griffin, Martin Sattler

**Affiliations:** ^1^ Department of Medical Oncology, Dana-Farber Cancer Institute, Boston, MA, USA; ^2^ Department of Medicine, Harvard Medical School, Boston, MA, USA; ^3^ Department of Biostatistics and Computational Biology, Dana-Farber Cancer Institute, Boston, MA, USA; ^4^ Karl Landsteiner University of Health Sciences, University Hospital Krems, Krems an der Donau, Austria; ^5^ German Cancer Research Center (DKFZ), Heidelberg, Germany; ^6^ Department of Medical Oncology and Therapeutics Research, City of Hope, Duarte, CA, USA; ^7^ Present address: LV Prasad Eye Institute, Bhubaneswar, India; ^8^ Present address: Center for Cellular and Molecular Medicine, Kyushu University Hospital, Fukuoka, Japan

**Keywords:** acute myeloid leukemia (AML), 6-phosphogluconate dehydrogenase (6PGD), cancer metabolism, FLT3, drug resistance

## Abstract

Acute myeloid leukemia (AML) cells are highly dependent on glycolytic pathways to generate metabolic energy and support cell growth, hinting at specific, targetable vulnerabilities as potential novel targets for drug development. Elevated levels of NADPH, a central metabolic factor involved in redox reactions, are common in myeloid leukemia cells, but the significance or biochemical basis underlying this increase is unknown. Using a small molecule analog that efficiently inhibits NADPH-producing enzymes, we found that AML cells require NADPH homeostasis for cell growth. We also found that inhibiting NADPH production through knockdown of 6-phosphogluconate dehydrogenase (6PGD) within the pentose phosphate pathway was sufficient to reduce cell growth and lactate production, a measure of metabolic reprogramming. Further, inhibition of 6PGD activity reduced NADH levels and enzymatic activity of the oxidized NADH-dependent sirtuin-1. Targeting 6PGD and NADPH production was sufficient to block growth of AML cell lines resistant to the chemotherapeutics daunorubicin and cytarabine. Importantly, stromal cell-mediated resistance to targeted inhibition of oncogenic FLT3 kinase activity by quizartinib was circumvented by 6PGD knockdown. Overall, these data suggest that the dependency of AML cells on NADPH to permit increased glycolytic flux creates a potential vulnerability of possible therapeutic benefit, since much of the enhanced production of NADPH is dependent on the activity of a single enzyme, 6PGD.

## INTRODUCTION

Acute myeloid leukemia (AML) is a genetically diverse cancer with a poor prognosis. Standard induction therapy with daunorubicin and cytarabine is plagued by the occurrence of drug resistance and does not lead to long-term remission in a large proportion of patients [1]. The only reliable treatment option that is an improvement over induction therapy is allogeneic hematopoietic stem cell transplantation, but it is not suitable for all patients [2]. Sequencing of the genetic landscape in AML has defined the major mutational pathways. AML patient specimens contain on average 3 to 5 recurrent mutations out of 15 mutations per sample [3]. The hope is that this knowledge will lead to the development of personalized medicine that will drastically improve outcome in AML. Of interest here are activating mutations in tyrosine kinases, which have shown to be good therapeutic targets in other malignancies. The most common target in AML is represented by recurrent mutations in the receptor tyrosine kinase FLT3, which occur in about one third of all patients [4]. A number of FLT3 kinase inhibitors have been shown to induce partial and transient remissions in clinical trials of relapsed AML patients when administered as single agents [5]. Current results demonstrate significant survival benefit for mutant FLT3 positive AML patients treated with the newly FDA Breakthrough Therapy designated midostaurin [6–8]. This in particular points toward the importance of FLT3 as a therapeutic target, at least in patients harboring FLT3 mutations. However, as drug resistance develops in some newly diagnosed AML patients and the majority of AML patients with advanced disease, other disease-driving mutations may exist and additional therapeutic approaches for mutant FLT3-positive AML are needed [9, 10]. An additional issue with therapeutic approaches in myeloid malignancies is that bone marrow stromal cells appear to provide a protective environment that cancels growth inhibitory effects by oncogenic tyrosine kinase inhibitors, in particular with respect to early disease-initiating cells. Pre-clinical models have been developed that capture some of these effects and they may help to identify novel approaches that can overwrite protective mechanisms [5, 11, 12].

Targeting reprogramming of glucose metabolism has emerged as an attractive and novel approach to developing new therapeutics in cancer treatment [13, 14]. The aberrant use of glucose and other carbon sources is part of a metabolic switch, leading to an increased glycolytic flux and elevated lactate production, even under aerobic conditions (Warburg effect) [15]. The pathways involved in glycolytic activities have been fairly well described in normal cells, but they may be intrinsically altered during oncogenic transformation. In myeloid malignancies, a hyper-active glucose metabolism is essential for increased mitochondrial electron transport chain activity [16, 17]. As a result, leukemic cells tend to produce an excess amount of reactive oxygen species during oxidative phosphorylation within the mitochondria. This oxidative stress is normally quenched by increased activity of the glutathione and thioredoxin scavenging systems to avoid toxicity [18]. NADPH plays a critical role as electron donor and scavenger of reactive oxygen in these reactions and its levels may be commonly elevated in leukemic cells [17]. However, the biochemical basis for this increase in AML is poorly understood.

6-phosphogluconate dehydrogenase (6PGD) and glucose-6-phosphate dehydrogenase (G6PD) within the pentose phosphate pathway are the major sources for NADPH in metabolically active cells [19]. Using cell-based models of AML, we found that blocking NADPH production with the NADP^+^ analog β-nicotineamide adenine dinucleotide 3’-phosphate was sufficient to inhibit growth of cells resistant to the chemotherapeutics daunorubicin and cytarabine. We further defined the potential for targeted therapy through reducing aberrant NADPH levels by inhibition of 6PGD function. The results show that knockdown of 6PGD not only partially inhibits its metabolic activity but also significantly reduces cellular NADPH levels. Reduced NADPH levels further affected NADH homeostasis, thus indirectly reducing NADH-dependent sirtuin activity. Targeting 6PGD cooperated in reducing cell growth in combination with daunorubicin and cytarabine treatment as well as in combination with inhibition of oncogenic FLT3 kinase activity by quizartinib. Importantly, inhibiting 6PGD activity or 6PGD-dependent mechanisms was sufficient to target drug-resistant cells, suggesting that 6PGD represents a potential vulnerability that could aid chemotherapy.

## RESULTS

### Myeloid leukemia cells require elevated NADPH levels for cell growth

**Figure 1:**
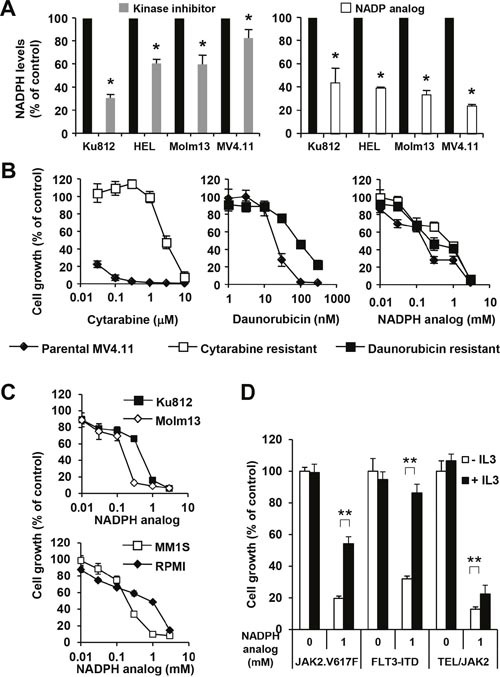
NADPH levels are required for increased growth **(A)** Changes in NADPH levels were measured in cellular extracts of KU812 (BCR-ABL), HEL (JAK2.V617F), Molm13 (FLT3-ITD) and MV4.11 (FLT3-ITD) in response to inhibition (24 h) of their respective oncogenic tyrosine kinase activities, including imatinib (6 μM), ruxolitinib (400 nM), and quizartinib (0.8 nM) (left) or in response to the NADPH analog β-nicotineamide adenine dinucleotide 3’-phosphate (KU812 - 1.4 mM; HEL, Molm13, MV4.111 - 0.4 mM) (right). *Significant differences (p<0.05; n=3) were observed between control and treated cells. Cell growth was measured (n=4) in **(B)** MV4.11 cells resistant to cytarabine (□) and daunorubicin (■) treated with either drug or the NADPH analog and compared to parental cells (♦) and in **(C)** KU812 (CML), Molm13 (AML), MM1S (multiple myeloma) and RPMI (lymphoma) treated with the NADPH analog, as indicated. **(D)** Untreated BaF3.EpoR.JAK2V617F (JAK2.V617F), BaF3.FLT3-ITD (FLT3-ITD) and BaF3.TEL/JAK2 cells were compared to cells treated with the NADPH analog, in the presence or absence of IL3. **Significant differences (p<0.005; n=4) were observed in response to IL3. Results were presented as mean ± SD.

Hematopoietic cells transformed by oncogenic tyrosine kinases are not only characterized by an increased glycolytic flux and a change in the metabolic profile but also by an elevation in NADPH levels as part of the cellular system required to maintain redox balance, to facilitate anabolic reactions and to fuel additional biochemical reactions. Consistent with our previous findings that hematopoietic cells expressing tyrosine kinase oncogenes can increase NADPH levels [17], we found that AML and CML patient-derived cell lines transformed by FLT3-ITD (MOLM-13 and MV4.11), BCR-ABL (KU812) or JAK2V617F (HEL), showed significantly reduced NADPH levels (p<0.05) in response to their respective kinase inhibitors (Figure 1A, left). Variability in the overall range of NADPH level (17.2% - 70.1% reduction) in response to the tyrosine kinase inhibitors points towards somewhat different mechanisms of activation in metabolic pathways. In order to define the significance of altered NADPH levels, we used the NADPH analog, β-nicotineamide adenine dinucleotide 3’-phosphate, as a chemical probe to target NADPH-dependent reactions. This inhibitor efficiently blocked NADPH dependent biochemical reactions *in vitro* (not shown) as well as reduced NADPH levels in MOLM-13 (33.8±3.8% of control), MV4.11 (23.8±1.6% of control), KU812 (43.7±12.6% of control) and HEL (39.3±1.0% of control) in cell-based assays (Figure 1A, right). As a major challenge in AML therapy is the resistance to standard chemotherapy, we therefore generated cytarabine and daunorubicin resistant AML cell lines to test the effect of the NADPH analog on cell growth. Whereas parental MV4.11 cells were at least 100 times more sensitive to cytarabine (IC_50_ < 0.03 μM) than cytarabine-resistant MV4.11 cells (IC_50_ = 3.22 μM), the difference between parental MV4.11 cells (IC_50_ = 20.01 μM) and daunorubicin-resistant MV4.11 cells (IC_50_ = 82.40 μM) was around four-fold (Figure 1B). In contrast, we observed considerably smaller differences in cell growth between parental MV4.11 cells (IC_50_ = 0.14 nM) and daunorubicin-resistant MV4.11 cells (IC_50_ = 0.31 nM) and cytarabine-resistant MV4.11 cells (IC_50_ = 0.74 nM) in response to the NADPH analog. The results demonstrate a crucial role for NADP/NADPH-dependent reactions in targeting cells resistant towards cytarabine and daunorubicine. Importantly, the efficacy of the NADPH analog was not limited to MV4.11 cells but was also found to reduce cell growth in KU812 (CML; IC_50_ = 0.37 mM), Molm13 (AML; IC_50_ = 0.22 mM), MM1S (multiple myeloma; IC_50_ = 0.17 mM) and RPMI-8226 (multiple myeloma; IC_50_ = 0.82 mM) cells (Figure 1C). Specificity of this approach was determined in factor-independent, transformed BaF3 cell lines, which allows for the comparison between normal (interleukin-3) signaling and oncogenic signaling (JAK2V617F, FLT3-ITD or TEL/JAK2) (Figure 1D). Treatment of these cells with interleukin-3 did not affect cell growth by itself in the absence of the NADPH analog. In contrast, the NADPH analog reduced cell growth and this effect could be rescued by interleukin-3 treatment (p<0.005) in BaF3.EpoR.JAKV617F cells (−IL3: 19.7±1.4% of control versus +IL3: 54.2±4.4% of control), in BaF3.FLT3-ITD cells (−IL3: 31.9±1.8% of control versus +IL3: 86.3±5.6% of control) and in BaF3.TEL/JAK2 cells (−IL3: 12.7±1.5% of control versus +IL3: 22.4±2.6% of control). Even though the NADPH analog functions as a chemical probe for these mechanistic studies, the results demonstrate a significant difference in the reliance on NADPH-dependent reactions between normal (IL3) and oncogenic signaling.

### NADPH and NADH levels are dependent on functional 6PGD expression

**Figure 2:**
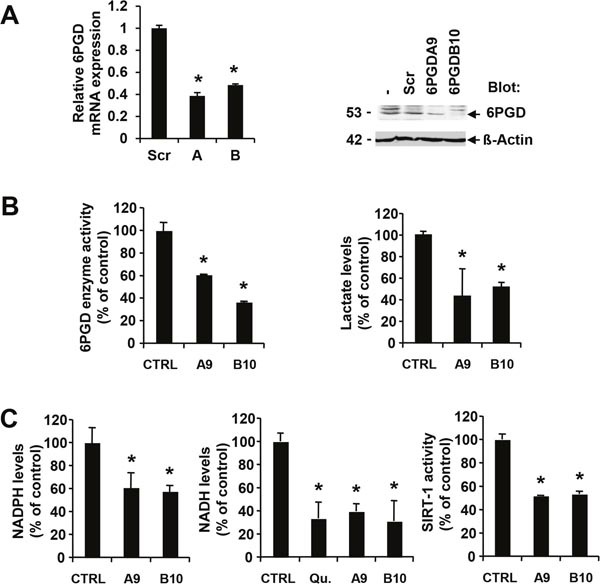
6PGD activity is required for optimal NADPH and NADH levels MV4.11 cells expressing scrambled shRNA (Scr) were compared to 6PGD-targeting shRNA. **(A)** Changes in either 6PGD mRNA expression (construct A and B) or protein expression (construct A, MV4.11-clone A9 or construct B, MV4.11-clone B10) were determined. **(B)** Changes in 6PGD enzyme activity or in lactate levels as well as **(C)** changes in NADPH levels, NADH levels or SIRT-1 enzyme activity were measured in cellular extracts and compared to controls (CTRL). In some experiments, cells were treated with 0.8 nM quizartinib (Qu.) for 24h. *Significant differences (p<0.05; n=3) were observed between control and 6PGD knockdown cells. Results were presented as mean ± SD.

The pentose phosphate pathway is thought to be the major source of reduced NADPH from its oxidized form through sequential oxidation of glucose-6-phosphate and 6-phosphogluconate by G6PD (glucose-6-phosphate dehydrogenase) and 6PGD (6-phosphogluconate dehydrogenase), respectively [19]. In order to determine the significance of this pathway and NADPH production for transformation, we specifically inhibited the second step of this reaction by targeted knockdown of 6PGD in MV4.11 cells. 6PGD expression was targeted with two different hairpins and reduction of 6PGD mRNA confirmed by semi-quantitative reverse transcription PCR (Figure 2A, left). Subclones of these cell lines with low 6PGD expression determined by immunoblotting were used for subsequent experiments (Figure 2A, right). Further, reduced levels of 6PGD were also reflected in reduced enzyme activity in cells containing 6PGD hairpin A (60.8±0.3% of control) or hairpin B (36.4±0.8% of control) (Figure 2B, left). Importantly, even though we only found partial but significant inhibition of 6PGD enzyme activity and the hairpins specifically targeted the pentose phosphate pathway through 6PGD, we also observed significantly reduced lactate levels with 6PGD hairpin A (43.2±25.4% of control) or hairpin B (51.6±4.7% of control) (Figure 2B, right). The data suggest that 6PGD is an integral component of metabolic reprogramming in these cells.

As a result of suppressed 6PGD activity, we also detected reduced NADPH levels in cells containing 6PGD hairpin A (61.0.8±12.7% of control) or hairpin B (57.7±4.9% of control) (Figure 2C, left). In addition to NADPH, we looked at levels of the related NADH in these cells as NADP is a product of the NAD kinase reaction [20]. Similar to NADPH, NADH levels were sensitive to inhibition of oncogenic FLT3 activity in MV4.11 cells by quizartinib (33.5.0.8±14.1% of control) (Figure 2C, middle). The changes in NADH levels were comparable to the reduction in cells containing 6PGD hairpin A (39.3±6.6% of control) or hairpin B (31.0±17.6% of control). To further define the biochemical consequences of altered NADH homeostasis, we measured sirtuin 1 (SIRT1) activity, which is dependent on oxidized NADH. SIRT1 is of interest in AML since it thought to be involved in drug resistance mechanisms [21]. Similar to the metabolic changes associated with 6PGD knockdown, we also observed a reduction in SIRT1 activity with 6PGD hairpin A (51.8±0.3% of control) or hairpin B (53.2±2.5% of control) (Figure 2C, right).

**Figure 3:**
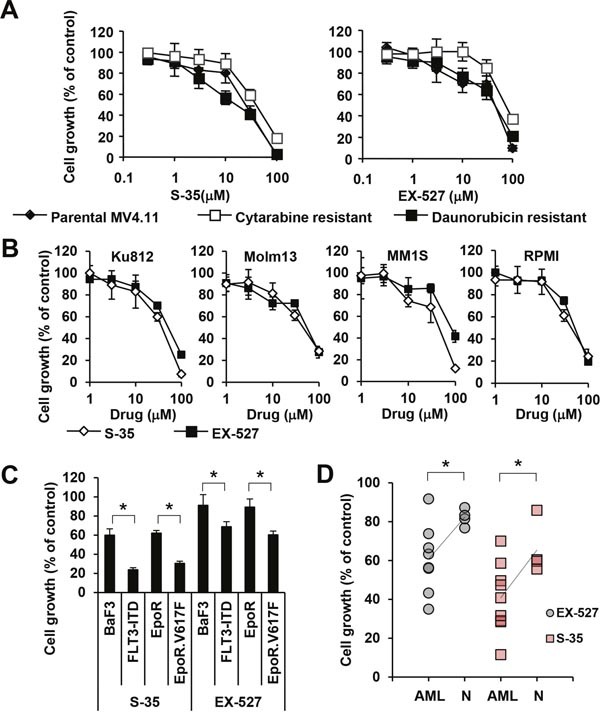
Efficacy of sirtuin inhibitors in various cell lines **(A)** Cell growth was measured (n=4) in MV4.11 cells resistant to cytarabine (□) and daunorubicin (■) that were treated with either the SIRT-1 inhibitor ‘compound (S)-35’ or the SIRT-1 inhibitor ‘EX527’ and compared to parental cells (♦). **(B)** Cell growth was measured (n=4) in KU812, Molm13, MM1S and RPMI-8226 treated with either compound (S)-35 **(◊)** orEX527 (■), as indicated. **(C)** BaF3 and BaF3.EpoR cells were compared to cells expressing either FLT3-ITD or JAK2V617F or **(D)** AML cells were compared to normal cells in response to 30 μM S-35 or 30 μM EX-527. Trend lines indicate the change in average. *Significant differences (p<0.05; n=3) were observed between control and transformed cells. Results were presented as mean ± SD.

SIRT1 is thought to be a key regulator of cellular metabolism and stress response genes, thus modulating a variety of cellular processes intrinsically related to transformation [22]. In order to define the role of SIRT1 downstream of 6PGD, we used the SIRT1 inhibitors (S)-35 and EX527 [23] to measure the effects on cell growth in parental MV4.11 cells as well as cells resistant to cytarabine and daunorubicin. Similar to the reduction of cell growth by the NADPH inhibitor (Figure 1B), both SIRT1 inhibitors reduced growth of MV4.11 cells in a dose-dependent manner (Figure 3A). The efficacy of both (S)-35 and EX527 in cytarabine-resistant MV4.11 cells (IC_50_ for (S)-35 = 12.8 μM; IC_50_ for EX527 = 36.5 μM) and daunorubicine-resistant MV4.11 cells (IC_50_ for (S)-35 = 37.1 μM; IC_50_ for EX527 = 74.8 μM) remained similar compared to the parental MV4.11 cells (IC_50_ for (S)-35 = 24.4 μM; IC_50_ for EX527 = 44.6 μM) and the results are similar to the efficacy in non-hematopoietic cells described by others, such as the IC_50_ of (S)-35 for reducing migration in MDA-MB-231 (breast cancer) = 20 μM [24] or the IC_50_ of EX527 for reducing growth in MCF-7 (breast cancer) = 50 μM [25]. Since metabolic reprogramming is a common mechanism in transformed cells, we also looked to see whether SIRT1 inhibition was effective in other myeloid leukemia cells (Ku812, Molm13) as well as non-myeloid hematopoietic malignancies (MM1S, RPMI-8226). Similar to the above results, (S)-35 and EX527 inhibited growth in a dose-dependent manner at similar concentrations for (S)-35 (IC_50_ in Ku812 = 31.2 μM, Molm13 = 41.4 μM, MM1S = 31.0 μM, RPMI-8226 = 42.3 μM) and for EX527 (IC_50_ in Ku812 = 46.4 μM, Molm13 = 45.2 μM, MM1S = 79.4 μM, RPMI-8226 = 45.5 μM) (Figure 3B). We further asked whether there are differences between oncogene-transformed and growth factor-dependent parental BaF3 cell lines with respect to inhibition with these inhibitors. Both S-35 and EX527 reduced cell growth in BaF3 cells (59.9±6.7% and 91.1±11.3% of control, respectively) and BaF3.EpoR (62.0±2.8% and 89.3±8.5% of control, respectively). However, growth was further reduced (p<0.05) by S-35 and EX527 in their FLT3-ITD (23.8±2.2% and 68.8±5.3% of control, respectively) and EpoR.Jak2V617F (30.5±2.3% and 60.3±4.0% of control, respectively) counterparts (Figure 3C). The selective inhibition of AML transformed cells was also confirmed in primary AML patient specimen (n=8) versus normal controls (n=4) with a significant reduction in cell growth (p<0.05) for S-35 (62.7% versus 82.2% of control) and EX-527 (40.8% versus 65.4% of control) (Figure 3D). These results do not exclude the possibility that other sirtuins or NADPH/NADH-dependent reactions are involved in transformation of these cells but highlight the involvement of the 6PGD/SIRT1 axis in cell growth.

### Targeting 6PGD cooperates with standard therapy and circumvents stromal protection

**Figure 4:**
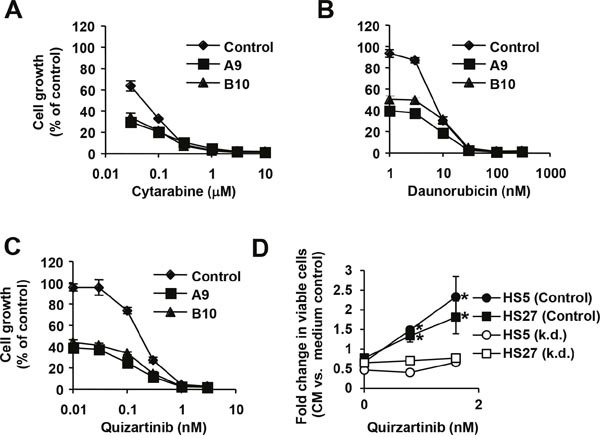
6PGD is required for increased growth Cell growth was measured (n=4) in MV4.11 cells containing 6PGD-targeting shRNA construct A (■), construct B (▲) and parental cells (♦) that were treated with **(A)** cytarabine, **(B)** daunorubicin or **(C)** the FLT3 inhibitor quirzatinib (Qu.), as indicated. **(D)** Cell growth was measured in a stromal cell model using either conditioned medium (CM) from HS5 or HS27 stromal cells. Fold changes in viable cells in response to conditioned medium were calculated relative to medium control-treated cells and measured in MV4.11 cells expressing scrambled shRNA (control) or 6PGD-targeting shRNA (k.d.) treated with different concentrations of quirzatinib. *Significant differences (p<0.05) were observed between control and treated cells. Results were presented as mean ± SD.

Even though cytarabine and daunorubicin are established and effective chemotherapeutics, neither of them provides a reliable treatment option. There is an ongoing search for better treatment options in AML. One alternative could be to improve upon existing therapy and combine these drugs with novel targeted therapies to further reduce growth of leukemic cells. We therefore sought to establish whether targeting 6PGD cooperates with these chemotherapeutics using drug-sensitive parental and 6PGD knockdown cells. Consistent with the incomplete knockdown and partial reduction in metabolism, growth of MV4.11 cells containing hairpin A (39.4 ±3.0 to 40.7 ± 1.7% of control) and hairpin B (39.8 ±2.7 to 44.1 ± 4.1% of control) was reduced compared to untreated MV4.11 cells containing scrambled shRNA (not shown and Figure 4A and 4B). Both, cytarabine and daunorubicin further reduced cell growth in a dose-dependent manner in all cells tested. We observed only marginal differences in growth between the two knockdown cell lines in response to either drug but found the 6PGD knockdown further reduced growth (Figure 4A and 4B). The data imply that 6PGD targeting does not interfere with the action of these chemotherapeutics and inhibiting this enzyme would cooperate with their effects on cell growth. Similar results were found with the FLT3 inhibitor quizartinib, which selectively inhibits oncogenic FLT3-ITD tyrosine kinase activity in MV4.11 cells (Figure 4C). Oncogenic FLT3 kinase activity is involved in metabolic reprogramming of leukemic cells [17, 26], yet targeting 6PGD also did not cancel the dose-dependent inhibition of cell growth by quizartinib. The results hint at a unique mechanism that may cooperate with anti-cancer drugs and improve the overall efficacy of targeted approaches.

Drug resistance is the main obstacle of successful leukemia treatment. In particular the stromal cell microenvironment is thought to protect early leukemic cells by providing stimuli that also support growth and viability of hematopoietic stem cells [11, 27]. We used an *in vitro* stromal cell model that has been well-characterized and includes conditioned medium from HS5 and HS27 stromal cell lines. These cells are capable of producing a variety of cytokines that are sufficient to support growth in drug resistance models of AML [28–31]. Stromal cell-conditioned medium does not enhance cell growth in MV4.11 cells but has a somewhat suppressive effect (HS5 at 0.67 ± 0.09 fold change and HS27 at 0.77 ± 0.04 fold change for MV4.11 cells; HS5 at 0.46 ± 0.03 fold change and HS27 at 0.65 ± 0.11 fold change for MV4.11 with 6PGD knockdown) (Figure 4D), possibly due to suppressive cytokines, nutrient exhaustion in the conditioned medium and the fact that the dominant growth-stimulating pathways are already activated. In the presence of quizartinib, cell growth is significantly suppressed (see also Figure 4C) The cytoprotective effects of the conditioned medium partially overwrites this growth suppression and leads to a net increase in viable cells, when compared to cells treated with control medium. As expected, there were no differences in the absence of quizartinib. The fold increases in viable cells relative to control-treated cells were less pronounced at 0.8 nM quizartinib (HS5 at 1.49 ± 0.09% fold change and HS27 at 1.34 ± 0.16 fold change) compared to a more potent concentration of 1.6 nM quizartinib (HS5 at 2.33 ± 0.52 fold change and HS27 at 1.81 ± 0.42 fold change) as a function of lower rescue potential relative to weaker growth suppression at lower drug concentrations. In contrast, the effect of stromal cell-conditioned medium was cancelled in MV4.11 with 6PGD knockdown and we did not observe significant increases between untreated cells (HS5 at 0.46 ± 0.03 fold change and HS27 at 0.65 ± 0.11 fold change), 0.8 nM quizartinib-treated cells (HS5 at 0.40 ± 0.02 fold change and HS27 at 0.70 ± 0.07 fold change) or 1.6 nM quizartinib-treated cells (HS5 at 0.66 ± 0.05 fold change and HS27 at 0.77 ± 0.11 fold change).

## DISCUSSION

The goal of current metabolomics research in cancer cells is to find strategies to selectively target AML cells exhibiting increased glycolytic flux with a relative sparing of normal cells [14]. We have previously shown that tyrosine kinase oncogenes in myeloid leukemias lead to a specific, yet broad increase in metabolite levels [32]. In particular, glucose metabolic pathways can distinguish AML from normal hematopoietic cells. The challenge is to identify the metabolic switches that control metabolic reprogramming. Our data have defined changes in NADPH levels in leukemic cells and identified 6PGD as one major enzyme that is specifically involved in this process. The role of 6PGD in NADPH production in itself is not surprising as it is together with G6PD one of the two major NADPH producing enzymes in the pentose phosphate pathway [19]. One unexpected finding here is the fact that partial inhibition (36-61% of control) of 6PGD enzyme activity is sufficient to reduce NADPH levels (58-61% of control) and growth (39-44% of control) to a similar extent, suggesting that 6PGD acts as a key regulator and points at a vulnerability within this pathway. Partial genetic deficiencies of this enzyme are largely tolerated by humans [33–35], suggesting that small molecule drugs that directly target 6PGD, or that lower pentose phosphate pathway activity, could provide therapeutic benefit in myeloid leukemias with minimal toxicity. In lung cancer models, functional 6PGD expression [36, 37] and acetylation [38] were associated with tumor growth. These data also hinted at a broader up-regulation of 6PGD activity in cancers, including in myeloid leukemias [38]. Thus, identifying potential metabolic switches in leukemic cells and validating these enzymes as potential therapeutic targets may also have implications for other cancers. However, considering the number of metabolic differences between normal and transformed cells and differences within the pool of patient specimen already found in our previous work [32], it is quite likely that metabolic dependencies may not be universal. For example, comparative proteome analysis found 6PGD to be specifically associated with AML subtype M2 but not M1 [39]. Whether this is also reflected in metabolic differences is unknown.

Our results provide an additional proof-of-concept for the approach of identifying and targeting metabolic switches and defining their role as a central mechanism in metabolic reprogramming and transformation. The results not only contribute to the understanding of metabolic networks and the interdependency of pathways therein, but also emphasize the potential of 6PGD and related molecules as targets to inhibit drug-resistant cell populations. As drug resistance is a major obstacle in AML, our results in particular with drug-resistant and drug-naïve models are of utmost clinical relevance. Mutant FLT3 has emerged as a convenient target in AML, but growth inhibition by selective inhibitors does not necessarily target the leukemic population that is protected by the stromal cell microenvironment. The protective mechanism appears to include at least in part stromal cell secreted cytokines that activate some of the very same pro-tumor pathways (i.e. phospho-STAT5) activated by oncogenic FLT3 [30].

Relative to normal hematopoietic progenitors, inhibition of 6PGD may not necessarily provide a wide enough therapeutic window to eradicate those leukemic cells on its own but it may be suitable to prevent growth- promoting signals within this niche. Our cytarabine drug resistance model, where we achieve higher levels of resistance compared to the daunorubicin-resistant cells, also demonstrates high efficacy of 6PGD as compared with traditional therapy with only marginal differences in IC_50_ values observed. It would be interesting to determine the effect of selective 6PGD inhibitors within this pathway as they become available. Nevertheless, previous studies have pointed to a metabolic weakness in cancer stem cells, which may make them more susceptible to metabolic approaches [40, 41]. It is likely that targeting 6PGD or related metabolic switches may cooperate with many chemotherapeutics and enhances toxicity in leukemic stem cells that may lead to their eradication.

It is not entirely clear from our data how 6PGD inhibition mediates growth suppression, but changes in NADH and lactate levels suggest that remodeling of metabolic pathways and substrate availability are part of an oncogenic network. For example, we have shown that knockdown of 6PGD alters NADH homeostasis and correlated with NAD^+^-dependent SIRT1 activity. SIRT1 is overexpressed in CD34^+^ AML [21], with a minimal role in normal hematopoietic cells [42] but involvement in metabolism, cell cycle control, metabolism, DNA repair and survival of cancer cells [22]. This is consistent with our data demonstrating reduced sensitivity towards sirtuin inhibitors in normal or growth-factor dependent models compared to AML or transformed cells. However, reduced NADH levels do not lead to concurrent reduced sirtuin activity [43]. SIRT1 can be regulated by a variety of mechanisms [44] and it would be interesting to sort out the complexity of metabolic changes caused by altered 6PGD activity that may contribute to this change. SIRT1 activity is of interest here since it has been implicated in the maintenance and drug resistance of FLT3-ITD-positive AML stem cells [21]. Indeed, our results are consistent with these findings and extend the efficacy of SIRT-targeted approaches to daunorubicin- and cytarabine-resistant cells. SIRT1 inhibition may also be an option in other hematologic malignancies that are plagued by drug resistance.

Similar to targeting 6PGD directly, the non-reactive small molecule NADPH analog, β-nicotineamide adenine dinucleotide 3’-phosphate, was effective in models of drug resistance and also inhibited growth of CML and multiple myeloma cancer cell lines. This approach may not be exclusive to inhibition of 6PGD and likely has some overlap with activities against other enzymes that can bind NADPH, even though we detected inhibitory activity of this compound against recombinant 6PGD (not shown). Despite the limitations of this approach, we found that factor-dependent growth stimulation was less sensitive to this inhibitor than oncogene-mediated growth, confirming increased dependency on NADPH-driven pathways in transformed cells. Thus, the results highlight the potential of inhibitors that interfere with NADPH homeostasis. Targeting NADPH levels may also significantly increase the efficacy of chemotherapeutics in leukemic cells that act through oxidative stress-inducing mechanisms. As leukemic cells tend to produce an excess amount of reactive oxygen species during oxidative phosphorylation within the mitochondria [16, 17], oxidative stress is balanced by increased activity of the glutathione and thioredoxin scavenging systems to avoid toxicity. NADPH plays a central role as an electron donor within these reactions, and reducing its levels will likely result in higher sensitivity of cancer cells toward stress-inducing events.

## MATERIALS AND METHODS

### Cell culture

Human cell lines KU812 (Ph+; CML), MOLM-13 (FLT3ITD+; AML) and MV4.11 (FLT3-ITD; AML), MM1S (multiple myeloma), RPMI-8226 (multiple myeloma), HS-5 (bone marrow stroma), HS-27 (bone marrow stroma) were grown in RPMI 1640 (GIBCO, life technologies, Carlsbad, CA) containing 10% fetal bovine serum (FBS; GIBCO, life technologies) and medium for HEL cells (JAK2.V617F+; erythroleukemia) contained additional sodium pyruvate (1mM; Invitrogen, Grand Island, NY). Murine BaF3 cells transformed by FLT3-ITD, TEL-JAK2 and BaF3 cells expressing the erythropoietin receptor (BaF3.EpoR) and expressing JAK2V617F were maintained in RPMI 1640 containing 10% fetal bovine serum. Parental BaF3 and BaF3.EpoR cells were maintained in medium containing additional WEHI-3B-conditioned medium as a source for interleukin-3 (IL3). Cell lines were from ATCC and were either used within 6 months or submitted for cell line authentication and were authenticated within 6 months of manuscript preparation through cell line short tandem repeat (STR) profiling (DDC Medical, Fairfield, OH and Molecular Diagnostics Laboratory, Dana-Farber Cancer Institute). All cell lines tested matched ≥80% with lines listed in the ATCC or DSMZ Cell Line Bank STR. Peripheral blood mononuclear cells (PBMCs) from normal individuals and mononuclear cells from AML patients were isolated by density gradient centrifugation through Ficoll-Plaque Plus (Amersham Pharmacia Biotech AB, Uppsala, Sweden) at 400g for 25 minutes, followed by two washes in PBS. Cells were then maintained in IMDM, supplemented with 20% FBS. Primary cells were obtained through written consent under approval of the Dana-Farber Cancer Institute Institutional Review Board. In some experiments, cells were treated with kinase inhibitors, including imatinib (Gleevec, Novartis, Basel, Switzerland), ruxolitinib (Active Biochemicals, Hong Kong, China), and quizartinib (Haoyuan Chemexpress, Shanghai, China), or SIRT-1 inhibitors, including compound (S)-35 (Santa Cruz Biotech., Dallas, TX) and EX527 (Santa Cruz Biotech.) or treated with β-nicotinamide adenine dinucleotide 3’-phosphate (Sigma-Aldrich, St. Louis, MO), cytarabine (Santa Cruz Biotechn.) and daunorubicin (Santa Cruz Biotechn.). Drug resistant MV4.11 cells were generated by outgrowth of cell population after selection with cytarabine (300 nM) and daunorubicin (17.5 nM).

### Cell growth

Cell growth was measured by trypan blue (Sigma-Aldrich) exclusion or with the CellTiter 96 Aqueous One Solution Cell Proliferation Assay reagent (Promega, Madison, WI). The CellTiter-Glo Luminescent Cell Viability Assay Kit (Promega, USA) was used to measure growth in BaF3 cell lines and primary cells, according to the manufacturer's instructions.

### Immunoblotting

Immunoblotting was performed as described previously using a standard chemiluminescence technique [45]. Rabbit monoclonal antibodies against 6-phosphogluconate dehydrogenase (ab129199, Abcam, Cambridge, MA) and mouse monoclonal antibodies against Δ-actin (12H8; Sigma-Aldrich) were used to document changes.

### Measurement of NADPH and NADH levels

NADPH and NADH levels were measured using NADPH and NADH calorimetric quantification kits (Abcam) according to the manufacturer's directions. Changes in levels were calculated relative to levels in extracts from untreated control cells.

### SIRT1 enzyme activity

Relative changes in SIRT1 activity were measured using a fluorometric SIRT-1 Activity Assay Kit (Abcam) according to the manufacturer's directions.

### Measurement of lactate production

Lactate production was measured in the supernatant of 1×10^6^ cells after incubation for 3h in serum-free medium using the Lactate Assay Fluorometric Kit (BioVision, Mountain View, CA).

### 6PGD enzyme activity assay

Cellular lysates were prepared in M-PER™ Mammalian Protein Extraction Reagent (Thermo Scientific, Waltham, MA) and 6PGD activities were determined by measuring the reduction of NADP+. Cell extracts were resuspended in a buffer containing 94 mM glycylglycine (pH 7.4 at 37°C) and 1.7 mM 6-phosphogluconate (Sigma). The reaction was started by the addition of 2.0 mM NADP+ (Sigma) and the increase in absorbance at 340 nm as a result of NADPH production was measured for 1 h. Changes in 6PGD activity were calculated relative to controls.

### Semi-quantitative real-time PCR

6-PGD gene expression was measured by semi-quantitative real-time PCR using specific primers (forward 5’-CGGATCATCCTCCTGGTG-3’; reverse 5’-ATGATGTCACCAGTATCCAACAA-3’) and human 60S acidic ribosomal phosphoprotein P0 (hRPLPO) control primers (forward 5′-GTGATGTGCAGCTG ATCAAGACT-3′; reverse 5’-GATGACCAGCCCAAAGG AGA-3’) to confirm efficient knockdown. Total RNA was extracted (RNeasy kit, Qiagen, Valencia, CA) to synthesize cDNA (Taqman Reverse Transcription Reagents, Applied Biosystems, Foster City, CA) for semi-quantitative real-time PCR (Power SYBR green PCR master mix, Applied Biosystems) using a 7500 Real-Time PCR System (Applied Biosystems).

### Targeted knockdown using lentiviral approaches

Knockdown of 6PGD was performed using two different lentiviral constructs (A and B; RNAi Screening Facility, Dana-Farber Cancer Institute) containing shRNA against 6PGD and compared to a scrambled control. Lentiviruses were generated by co-transfecting HEK293T cells with viral packaging vectors pMD2.G and pCMVΔ8.91 as well as shRNAs using the TransIT (Mirus, Madison, WI) reagent. KU812 cells were infected in the presence of polybrene (5μg/mL; Millipore, Temecula, CA) and selected for one week in medium containing puromycin (1μg/mL; Sigma).

### Statistical analysis

For statistical comparison between test and control groups, the Student's *t*-test or the Welch's *t*-test (experiments involving stromal cell-conditioned medium and quizartinib only) were used. Changes were calculated as the percentage change relative to the average of the control. Error bars represent standard deviation (SD) of at least three independent experiments.
